# Synergistic Interaction in the Analgesic-Like Effects of Maqui Berry and Citrus Is Antagonized by Sweeteners

**DOI:** 10.3390/nu13072466

**Published:** 2021-07-19

**Authors:** Vicente Agulló, María Eva González-Trujano, Alberto Hernandez-Leon, Erika Estrada-Camarena, Francisco Pellicer, Cristina García-Viguera

**Affiliations:** 1 Laboratorio de Neurofarmacología de Productos Naturales, Dirección de Investigaciones en Neurociencias, Instituto Nacional de Psiquiatría Ramón de la Fuente Muñiz, Calz. Mexico-Xochimilco 101, Col. San Lorenzo Huipulco, Tlalpan, Ciudad de Mexico 14370, Mexico; vagullo@cebas.csic.es (V.A.); albertoh-leon@hotmail.com (A.H.-L.); pellicer@imp.edu.mx (F.P.); 2 Grupo Calidad, Laboratorio de Fitoquímica y Alimentos Saludables (LabFAS), Bioactividad y Seguridad, Departamento de Ciencia y Tecnología de Alimentos, CEBAS-CSIC, Campus de Espinardo 25, 30100 Murcia, Spain; 3 Laboratorio de Neuropsicofarmacología, Dirección de Investigaciones en Neurociencias, Instituto Nacional de Psiquiatría Ramón de la Fuente Muñiz, Calz. Mexico-Xochimilco 101, Col. San Lorenzo Huipulco, Tlalpan, Ciudad de Mexico 14370, Mexico; estrada@imp.edu.mx

**Keywords:** anthocyanins, antinociception, citrus, polyphenolic compounds, sweeteners

## Abstract

Although physiologically pain has a protective function, in many diseases, it is one of the most prominent symptoms. Today, new trends are focused on finding more natural alternatives to conventional treatments to alleviate it. Thereby, the purpose of this investigation was to obtain preclinical data of the antinociceptive properties of a lyophilized obtained from a newly designed maqui–citrus beverage alone and added with different sweeteners. To achieve this objective, maqui berry and citrus pharmacological activity were studied separately, as well as the interaction of both ingredients. In addition, due to the controversy generated regarding the intake of sugars, related to different metabolic diseases, the influence of different sweeteners (stevia, sucralose, or sucrose) was studied to determine their possible influence on the bioactive compounds of this product. For the attainment of our goals, a pharmacological evaluation, using the 1% formalin test, a nociceptive pain model in mice, was performed by using a sub-efficacious dosage of Maqui (25 mg/kg, i.p.) alone and combined with citrus, and then compared with the effects obtained in the presence of the different sweeteners. As a result, the antinociceptive response of the maqui was synergized in the presence of citrus in the neurogenic and inflammatory phases of the formalin test. However, this response was partially or totally reduced in the presence of the sweeteners. Our study gives preclinical evidence that a combination of maqui and citrus might exert beneficial actions to relieve pain, whereas the presence of sweeteners could reduce or avoid it.

## 1. Introduction

Pain has a physiological protective role by following several mechanisms that lead to the inhibition of the source of pain itself and in cases of injury, infection and/or trauma, it is a remarkable symptom [1].

Based on their mechanism of action, the classification of pharmacological treatments for pain relief includes analgesics that act at central or peripheral nervous systems. Unfortunately, adverse effects from moderate to severe are very common and limit this optional resource for pain relief [2].

In order to contribute to responding to such demand, we studied a new previously own-developed maqui–citrus beverage [3], rich in bioavailable bioactive compounds, with anti-inflammatory and antioxidant activity [4,5,6,7], which we look to propose as a natural treatment to relieve pain.

*Aristotelia chilensis* (Mol.) Stuntz (Elaeocarpaceae), commonly named maqui, is a wild berry that grows in the south of Chile and western Argentina, widely used in native traditional herbal medicine, due to its analgesic and anti-inflammatory properties, among others [8]. A previous and preliminary pharmacological study already demonstrated its dose-dependent antinociceptive activity, not only at central but at peripheral levels, reducing nociceptive and inflammatory phases in a nociceptive pain model in mice [9].

Citrus fruits are considered good sources of nutrition containing considerable quantities of ascorbic acid (also known as vitamin C) and high concentrations of flavonoids, mainly flavanones (95%), which are bioactive metabolites with demonstrated health benefits, such as the protective role against oxidative damage. Citrus flavonoids might exert low or no cytotoxicity on healthy cells. The importance of citrus in pain relief in diseases involving neurodegeneration and the central nervous system (CNS) has been associated with the analgesic effects of their flavonoids since they can cross the blood–brain barrier (BBB) [10]. Because of this, a combination of citrus with maqui berry might be the base of new bioactive beverages with potential activity to face different diseases, such as pain and neurological system disorders. This statement is based on previous studies [4,5,6,7], focused on the intake of a recently developed maqui–citrus beverage, with different added sweeteners, in which the bioaccessibility and bioavailability of the bioactive compounds, as well as their effects on glycemic response in overweight subjects, have been demonstrated. However, the activity against different diseases has not yet been described.

Thereby, as pharmacological properties of maqui in relieving pain have been previously described [9], the purpose of this study is to provide preclinical information regarding the pharmacological effects of a combination of maqui–citrus with different sweeteners added for future food formulations and as an alternative to common over-the-counter (OTC) pain relievers.

## 2. Materials and Methods

Maqui New Life S.A. (Santiago de Chile, Chile) supplied the maqui berry powder as a soluble freeze-dried of the entire fruit, rich in anthocyanins (1.6% of total weight of powder that represented over 85% of the total phenolic compounds). Cítricos de Murcia S. L. (Ceutí, Spain) and AMC Grupo Alimentación Fresco y Zumos S. A. (Espinardo, Spain) provided the citrus juices. Sucrose, stevia, and sucralose were provided by AB Azucarera Iberia S.L. (Madrid, Spain), AgriStevia S.L. Stevia Rebaudiana Extract 98% (Molina de Segura, Spain), and Zukan (Murcia, Spain), respectively. The combination of maqui berry powder (MB) and citrus juice was processed as previously described [5,6,7]. Briefly, both ingredients were mixed to obtain the base mixture (MB + Citrus, pH = 3.6). Then, the three selected sweeteners were added to obtain the different sweetened samples in different proportions, 4 mg per 100 mL for stevia (pH = 3.7) and sucralose (pH = 3.6), and 7.5 g per 100 mL for sucrose (pH = 3.9). Citrus and maqui–citrus beverages were freeze-dried for storage purposes and freshly redissolved in distilled water previously to mice administration.

### 2.1. Analysis of Anthocyanin and Flavanone

Cyanidin (Cy) and delphinidin (Dp) 3-O-glucoside chloride, were purchased from TransMIT (Geiben, Germany); hesperidin, eriocitrin and narirutin from Merck (Darmstadt, Germany), formic acid from Fisher-Scientific (Loughborough, UK) and methanol (LC-MS Chromasolv) from Honeywell/Rieden-de-Haen (Seelze, Germany). Ultrapure water from a Milli-Q Advantage A10 ultrapure water purification system (Merck Millipore, Darmstadt, Germany) was used in preparation of all standards of natural products.

Identification and total phenolic content of the maqui berry powder were performed through the method previously reported in the literature [3].

### 2.2. Pharmacological Study

#### 2.2.1. Animals

Male Swiss Webster mice (25–30 g) bred in the vivarium of Instituto Nacional de Psiquiatría “Ramón de la Fuente Muñiz” were used in this study. All animals had ad libitum access to tap water and Purina rat chow (Lab Chow, 5001, Purina, St. Louis, MO, USA) throughout the experiments. Experimental rooms were maintained under a controlled light–dark cycle (12 h:12 h) with a temperature of 21–23 °C. All tests were performed according to the Official Mexican Norm for care and handling animals (NOM-062-ZOO-1999), the International Association for the Study of Pain (IASP) and approved by our institutional ethics committee (CONBIOETICA-09-CEI-010-20170316).

#### 2.2.2. Reagents and Drugs

Tramadol (TRA) at a pharmaceutical grade was purchased from Grünenthal México S.A. de C.V. (Mexico City, Mexico); a 37% formaldehyde solution was purchased from J.T. Baker (Phillipsburg, NJ, USA). The maqui berry powder (25 mg/kg) in the presence of the citrus juices or the different sweeteners, was freshly prepared with distilled water on the day of the experiment. Pharmacological evaluation was performed by using an intraperitoneal (i.p.) administration to improve the bioavailability of constituents according to a preliminary comparison of the dose–response effects between enteral and parenteral administration of the redissolved maqui–citrus freeze-dried beverage [9]. A volume of 0.1 mL/10 g of body weight volume for i.p. administration. Distilled water was administered to mice in the control group.

#### 2.2.3. Experimental Design to Explore the Antinociceptive Activity

In order to evaluate the antinociceptive activity of treatments, eight groups (*n* = 6 mice) were evaluated as follows: (1) Control group received the vehicle consisting of distilled water; (2) Tramadol (TRA, 30 mg/kg, i.p., analgesic reference drug); (3) maqui berry powder alone (MB, 25 mg/kg, i.p.); (4) citrus juice alone (25 mg/kg, i.p.); (5) a combination of maqui berry and citrus (MB + citrus); and the combinations of MB + citrus plus a sweetener: (6) Stevia, (7) Sucralose, or (8) Sucrose. The amount of citrus and sweeteners for the combination with MB was in accordance with the formulation of the previously developed MB + citrus beverages [3]. The dose of treatments was based on our preliminary acute studies in healthy volunteers [5,6,7], where the bioavailability of anthocyanin and flavanone was analyzed after the intake of 330 mL of a beverage containing 3.3 g of *A. chilensis* (54.75 mg of anthocyanins) and 214.5 mL of citrus (28.75 mg of flavanones) [3], and in the results attained in our antinociceptive study in maqui [9]. Treatments were injected by i.p. route of administration 30 min before the intraplantar injection of the 1% formalin nociceptive algogenic. It is important to mention that acidic solutions of experimental treatments did not produce interference in the nociceptive behavior in the formalin test in mice. This fact was corroborated by using a control group receiving an intraperitoneal administration of acidic saline solution with a similar pH = 3.6 and with another group where the well-known algogenic substance such as 0.5% acetic acid was used (pH = 2.6), which produced a significant number of writhes but without alteration of the nociceptive behavior in the formalin test (Supplementary Material, Figure S1). These data reinforce the genuine antinociceptive response of maqui plus citrus treatment in the formalin test observed in this study.

Formalin test—Animals were allowed to habituate to the testing environment for 30 min (a 30 × 12 × 13 cm clear Plexiglas box fitted with mirrors to enable a total panorama of the nociceptive response). Then, mice were administered with one of the eight previously mentioned treatments and thirty minutes later, 20 µL of formalin was injected subcutaneously into the plantar surface skin of the right hind paw of the mouse using a microsyringe with a 30-gauge needle. The mouse was then returned into the Plexiglas box for the immediate observation of the nociceptive behavior to build a temporal course curve of the time spent licking the injected paw. Behavioral response was counted 1 min each 5 min in a half-hour period of registration after formalin injection as follows: T1: 0–1 min, T2: 5–6 min, T3: 10–11 min, T4:15–16 min, T5: 20–21 min, T6: 25–26 min, T7: 30–31 min. This test includes two periods of high activity: the first one covered from the moment of injection to 10 min later (T1–T3), known as phase I. The second period, named phase II from 10 min to 30 min (T3–T7) after the 1% formalin injection, is called the later or the inflammatory phase.

Data obtained in the temporal course curves allowed determining the time-interval of neurogenic nociception at central level (phase I) and the peripheral or inflammatory stage (phase II). The area under the curve was calculated for both the neurogenic and inflammatory phases by using the trapezoidal rule to identify the significant antinociceptive response of treatment combinations.

### 2.3. Statistical Data Analysis

Statistical differences in the time course curves were determined by repeated-measures two-way ANOVA followed by Bonferroni’s post-hoc test. Statistical significance in the nociceptive response, expressed as the area under the curve (AUC) in bars was established by using one-way ANOVA followed by Tukey’s post-hoc test. GraphPad Prism software, version 8.0.2. (GraphPad Software INC, La Jolla, CA, USA) was used for statistical procedures. Level of significance was set to 5% (*p* < 0.05). Results are expressed as the mean ± standard error of the mean (S.E.M.).

## 3. Results

### 3.1. Phenolic Content of Maqui Berry Powder and Citrus

Regarding the anthocyanin’s composition of the maqui berry powder, eight different anthocyanins were characterized in a total of 16.59 mg/g maqui powder. Delphinidin derivates were the most abundant (≈83%) followed by cyanidin ones (Table 1).

With respect to flavanone composition of the citrus beverage (total concentration: 1.56 mg/g citrus), individual compounds were found in the following decreasing concentration order: hesperetin 7-*O*-rutinoside > eriodictyol 7-*O*-rutinoside > naringenin 7-*O*-rutinoside > *O*-tri-glycosyl-naringenin (Table 2).

### 3.2. Synergistic Antinociceptive Activity of Maqui Berry and Citrus

**Figure 1:**
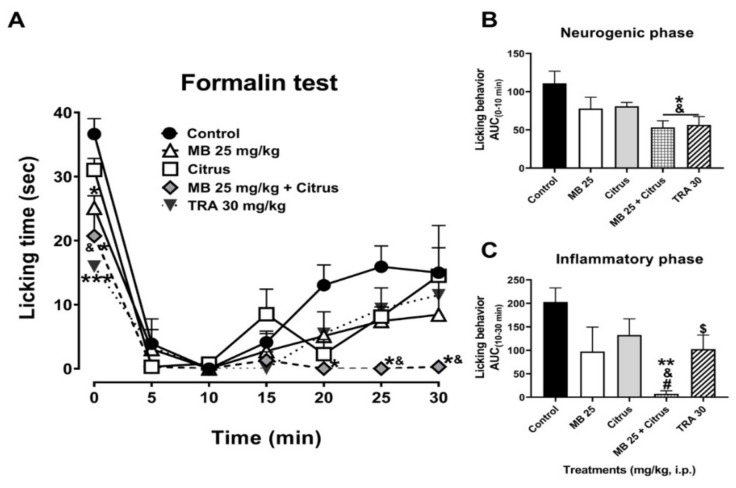
(**A**) Time course curves of the antinociceptive effect of maqui (MB) and citrus alone and combined, as well as tramadol (TRA, a reference drug, 30 mg/kg) after intraperitoneal administration. Two-way ANOVA followed by Bonferroni’s post-hoc test. * *p* < 0.05 and *** *p* < 0.001 vs. control group, & *p* < 0.05 vs. citrus group. *n* = 6 repetitions. Antinocicepive response of treatments expressed as the area under the curve (AUC) in the neurogenic ((**B**), 0–10 min) and inflammatory ((**C**), 10–30 min) phases of the formalin-induced nociception in mice. One-way ANOVA followed by Tukey’s post-hoc test. * *p* < 0.05 and ** *p* < 0.01 vs. control group, & *p* < 0.05 vs. citrus group, # *p* < 0.05 vs. MB group. Student’s t test, $ *p* < 0.05 vs. control group. *n* = 6 repetitions.

Tramadol (TRA, 30 mg/kg, i.p.), as a partial opioid reference analgesic drug, produced significant inhibition of licking behavior in the neurogenic phase as shown in the temporal course curve (Figure 1A) and dose–response graphic (Figure 1B). A dosage of citrus alone produced a non-significant reduction in neurogenic nociceptive behavior. Meanwhile, a significant reduction in the nociceptive behavior was observed in the MB (25 mg/kg, i.p.) administration that almost reached the significant effect of the mixture observed in the first minute after the algogenic agent injection (Figure 1A). Regarding the inflammatory phase, both MB (25 mg/kg, i.p.) and citrus separately showed a non-significant reduction (Figure 1A). In marked contrast, the combination of these two treatments produced a total reduction in the nociceptive behavior in all the period registered for this phase (Figure 1A) (Treatment F_4,25_ = 7.58, *p* = 0.0004; Time F_2.924,73.09_ = 42.33, *p* < 0.0001; Interaction F_24,150_ = 1.66, *p* = 0.0355).

The significant nociceptive response obtained with the combination of MB + citrus, both at sub-efficacious dosage in the neurogenic phase, resembled that observed with the reference drug at 30 mg/kg (F_4,25_ = 3.95, *p* = 0.013), clearly observed in the integration of the area under the curve where both tramadol (t = 2.38, df = 10, *p* = 0.039) and MB + citrus showed a difference in comparison to the vehicle (Veh) and individual administration of citrus (Figure 1B).

A complete significant inhibition in licking behavior was observed in the inflammatory phase in mice receiving the combination of MB (25 mg/kg) + Citrus in comparison to all the other treatments (F_4,25_ = 4.36, *p* = 0.0082) (Figure 1C).

### 3.3. Antagonistic Action of Sweeteners on the Antinociceptive Activity of Maqui Berry and Citrus

**Figure 2:**
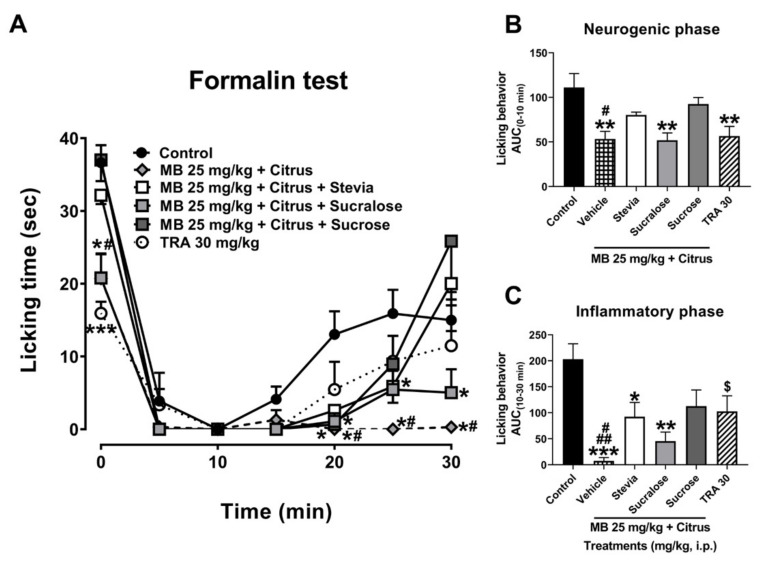
(**A**) Time course curves of the antinociceptive effect of maqui–citrus (MB + citrus) alone and added with sweeteners stevia, sucralose, or sucrose. Tramadol (TRA, a reference drug). Two-way ANOVA followed by Bonferroni’s post-hoc test, * *p* < 0.05 and *** *p* < 0.001 vs. control group, # *p* < 0.05 vs. stevia and sucrose in the 1st, 25 and 30 min, and sucralose from 25 min, after formalin test in mice. Antinocicepive response of treatments expressed as the area under the curve (AUC) in the neurogenic ((**B**), 0–10 min) and inflammatory ((**C**), 10–30 min) phases. One-way ANOVA followed by Tukey’s post-hoc test. * *p* < 0.05, ** *p* < 0.01, and *** *p* < 0.001 vs. control group, # *p* < 0.05 vs. stevia and sucrose in neurogenic phase. # *p* < 0.05 vs. sucralose and ## *p* < 0.01 vs. stevia and sucrose group in inflammatory phase. Student’s t test, $ *p* < 0.05 vs. control group (t = 2.38, df = 10, *p* = 0.039). *n* = 6 repetitions.

In the administration of MB + citrus with sweeteners, the significant antinociceptive response of the MB + citrus was avoided in the presence of sweeteners stevia and sucrose, whereas it was not modified in the presence of sucralose, as it was observed in the first minute of the neurogenic stage in the temporal course curve (Figure 2A). No changes were observed in the first 10 min of the inflammatory phase in the presence of each sweetener evaluated with the mixture. However, the maximal antinociceptive effect of MB + Citrus was lost after this time, again in a total manner in the presence of stevia or sucrose, and partially by sucralose as observed at the end of the evaluation of the inflammatory phase (Figure 2A, Treatment F_5,30_ = 11.35, *p* < 0.0001; Time F_2.508,75.24_ = 71.42, *p* < 0.0001; Interaction F_30,180_ = 2.98, *p* < 0.0001).

In all the AUC of the neurogenic phase, the antinociceptive response of the mixture MB 25 + Citrus was antagonized in the presence of the sweeteners such as stevia and sucrose, without alteration in the case of the presence of sucralose (Figure 2B) (F_5,30_ = 6.29, *p* = 0.0004). Meanwhile, in the inflammatory phase, a partial inhibition in the antinociceptive effect of MB 25 + Citrus was observed in the presence of sucralose (from *p* < 0.001 to *p* < 0.01). This inhibition was more evident when mixture was combined with stevia (from *p* < 0.001 to *p* < 0.05), and sucrose from (*p* < 0.001 to *p* < 0.05) (Figure 2C) (F_5,30_ = 6.93, *p* = 0.0002).

## 4. Discussion

In our work, a maqui berry + citrus mixture with high anthocyanin and flavanone concentration demonstrated a synergistic antinociceptive activity interaction in comparison to the effect obtained with maqui berry and citrus separately. The presence of sweeteners in this mixture blocked this beneficial interaction observed in the chemical pain model induced in the formalin test in mice using an intraperitoneal administration.

The administration of substances to laboratory animals requires careful consideration and planning to optimize the delivery of the agent to the animal. Although intraperitoneal delivery is considered a parenteral route of administration, the pharmacokinetics of substances administered intraperitoneally are more similar to those seen after oral administration, because the primary route of absorption is into the mesenteric vessels, which drain into the portal vein and then pass through the liver [11]. Therefore, these route administration results are very helpful to optimize the efficacy of treatments and a common strategy for preclinical pharmacological studies.

The formalin test was used in this study as an acute and tonic stimulus to elicit the nociceptive behavior (licking time) in the neurogenic and inflammatory phases. The neurogenic phase is thought to be triggered by the direct activation of C-fibers due to the peripheral stimulus, whereas the inflammatory phase reflects the nociception produced by the combination of acutely inflamed tissue and functional changes in the dorsal horn of the spinal cord [12]. It is well known that centrally acting drugs, such as opioids, inhibit both phases because they primarily act by modulating the descending inhibitory nociception pathway. Drugs with peripheral actions such as non-steroidal anti-inflammatory drugs, which block the prostaglandin synthesis, reduce nociception by inhibiting only the inflammatory phase [13].

In accordance with our objective, a significant antinociceptive effect was produced in both phases of the formalin test in the presence of a sub-effective dosage of maqui berry combined with citrus, suggesting pharmacological actions at central and peripheral levels. The neurological activity of maqui berry alone has been mainly related to its high anthocyanin concentration, whose intact or derived metabolites, mainly of delphinidin nature, are capable of crossing the blood–brain barrier [14]. Previous reports have demonstrated that these anthocyanins are bioavailable for humans [5,6,7], and they could be discovered intact in the brain, among other tissues [15]. Stabilization of delphinidin, in a formulation for systemic administration, reversed mechanical and thermal hyperalgesia, as well as local inflammation, in part because of its capacity to scavenge superoxide anion radicals with an inhibitory concentration of 70 ± 5 µM [16]. Anthocyanins can also modulate the Nrf2 pathway to mitigate oxidative stress or neurodegeneration [17]. The high content of anthocyanin in maqui berry [9,18,19], like the abundant derivative of the delphinidin compound, might influence the antinociceptive activity of this species through the intervention in antioxidant and anti-inflammatory pathways [20]. Delphinidin and cyanidin, among the major anthocyanidins in berries, are powerful active ingredients that have potential antioxidant activity [16]. A fraction of Vaccinium angustifolium Ait (wild blueberry) containing a high concentration of anthocyanins including delphinidin derivates showed significant inhibition of cyclooxygenase-2 (COX-2), inductible nitric oxide synthase (iNOS), and interleukin-1β (IL-1β), with less influence on the IL-6, markers of acute inflammatory response [21]. In addition, these anthocyanidins have the ability to inhibit inflammatory and pain mediators such as the expression of tumor necrosis factor-alpha (TNF-α) [22]. All these mechanisms together could influence the antinociceptive activity of maqui berry in the neurogenic and inflammatory phases at both central and peripheral levels.

The presence of citrus produced a synergistic interaction with maqui berry by improving its antinociceptive properties since a significant response was observed when they were administered in combination compared to the individual administration. It is known that the healthy properties of citrus, mainly attributed to its antioxidant capacity, are related to its high vitamin C content and flavonoids [23]. Citrus fruits are especially rich in flavanones such as hesperidin, narirutin and eriocitrin. Flavonoids are natural compounds that target multiple steps in the inflammatory pathway as compared to monotargeted synthetic anti-inflammatory drugs [24]. The flavonoid naringenin administered by oral and intraperitoneal route of administration significantly reduced the nociceptive response in several models of pain in mice, including the formalin test, by modulating several mechanisms such as the NO-cyclic GMP-PKG-ATP pathway and glutamatergic and opiodergic neurotransmission without the presence of common adverse effects observed in clinic analgesic drugs [25,26]. Similarly, the antinociceptive activity of rutin was due to the PAG circuitry involving the participation of opioid receptors [27]. The main citrus flavonoids can also cross the blood–brain barrier [28]; thus, they are promising candidates in neurodegeneration research and as constituents of brain foods due to their potential activity as reactive oxygen species (ROS) scavengers [29]. The ROS might be produced by excessive production of chemokines and cytokines, which are regulatory proteins under normal physiological conditions, but in excess, they disrupt the gradient balance of the gradient, generating chronic inflammation. When administered individually, flavonoids produced antihyperalgesic effects in different kinds of pain models. In the case of hesperidin, its antihyperalgesic effects in neuropathic pain have been related to the presence of cytokines concentrations (TNF-α, IL-1β and IL-6) in both peripheral and central tissues [10]. Both hesperidin and diosmin were detected in rat brains and significantly reduced behavior in neuropathic pain; when they were administered in combination, they improved the response mediated by a partial modulation of D2, GABA_A_, and opioids, but not by 5-HT_1A_, receptors [30], reinforcing the idea that a combination of neuroprotective natural products is better than their individual action. Combinations are not exclusive to natural products, as they can also be mixed with analgesic drugs to reduce effective doses and some adverse effects. Metamizole is used to relieve visceral pain, but its use is limited by its adverse effects. An alternative to improve its efficacy with lower doses was to combine it with a natural product such as hesperidin [31]. A synergistic interaction was also observed when antinociceptive doses of hesperidin were combined with those of ketorolac, another analgesic drug widely used in the clinic, producing 15 combinations of mainly additive and supra-additive responses [32]. These results are consistent with the synergistic activity observed in the present study when maqui berry and citrus are combined, since hesperidin is the main flavanone of the maqui–citrus beverages analyzed.

Naturally sourced sweeteners are generally believed to be safe. However, it is known that there is no health advantage in the intake of any particular type of sugar added to nutriments. In fact, they can lead to health problems due to poor nutrition. These substances are common in beverages, but they need to be evaluated since the beneficial health effects of these beverages could be inhibited in the presence of some sweeteners. High-intensity artificial sweeteners, heavily promoted by the food industry, are among the most controversial food additives for suspected adverse effects such as dermatological problems, headaches, mood variations, behavior changes, respiratory difficulties, seizures, allergies, and cancer [33].

Interestingly, in our study, the presence of sweeteners avoided the antinociceptive effects achieved with the combination of maqui berry with citrus. Sucralose produced a partial inhibition of the significant antinociceptive response, while sucrose followed by stevia might completely inhibit the beneficial synergistic activity. We cannot discard that these effects might be associated with pharmacodynamic mechanisms, but also with a pharmacokinetic influence since sucralose provided the greatest absorption value in humans for most of the maqui berry metabolites, followed by stevia and with a minor influence of sucrose after the ingestion of a new maqui–citrus-based beverage supplemented with these sweeteners. These results suggest that non-caloric sweeteners are better alternatives than a high caloric sweetener to avoid the augmented risk of several metabolic disorders [7,34] (Reference [35] is cited in the Supplementary Materials), but also as an alternative to painful effects as was observed in this preclinical study.

Nevertheless, one aspect that might be considered is that the oral consumption of stevia, sucralose and sucrose does not lead to absorption of the intact compounds into the blood. Therefore, the inhibitory effects of intact stevia, sucralose or sucrose on the beneficial synergy of the maqui berry and citrus, which are reported in this paper, might not occur during oral consumption. This will require further testing.

## 5. Conclusions

The present results provide preclinical evidence that the combination of maqui berry plus citrus produces synergism for beneficial actions in pain therapy. However, in the presence of sweeteners, these properties could be reduced or avoided. In this study, an intraperitoneal injection was used as a strategy to improve the bioavailability of treatments avoiding drastic reduction and subtherapeutic action likely observed in oral administration. However, it would be interesting to explore and compare efficacies by using this route of administration in future studies. In addition, upcoming research focused on the biological targets is required to understand the peripheral and central antinociceptive effects of maqui berry alone or in the presence of citrus. Our findings make this newly developed beverage a potentially healthy option for pain relief.

## Figures and Tables

**Table 1:** Anthocyanin composition of maqui berry powder [9].

Compound		Anthocyanins (mg/g Maqui)
Delphinidin 3-*O*-sambubioside-5-*O*-glucoside		4.01 ± 0.01
Delphinidin 3,5-*O*-diglucoside		3.51 ± 0.02
Cyanidin 3,5-*O*-diglucoside + Cyanidin 3-*O*-sambubioside-5-*O*-glucoside		1.76 ± 0.01
Delphinidin 3-*O*-sambubioside		1.90 ± 0.02
Delphinidin 3-*O*-glucoside		4.29 ± 0.04
Cyanidin 3-*O*-sambubioside		0.05 ± 0.00
Cyanidin 3-*O*-glucoside		1.07 ± 0.00
	Total	16.59 ± 0.04

**Table 2:** Flavanone composition of citrus.

Compound		Flavanones (mg/g Citrus)
*O*-tri-glycosyl-naringenin		0.12 ± 0.01
Eriodictyol 7-*O*-rutinoside		0.17 ± 0.01
Naringenin 7-*O*-rutinoside		0.43 ± 0.01
Hesperetin 7-*O*-rutinoside		0.83 ± 0.01
	Total	1.56 ± 0.02

## Data Availability

The data presented in this study are available on request from the corresponding author.

## Supplementary Materials

The following are available online at https://www.mdpi.com/article/10.3390/nu13072466/s1, Figure S1: (A) F_5,12_ = 194.3, *p* < 0.0001 comparison of all the treatments vs. 0.5% acetic acid group in writhing behavior. Acidic s.s. as a control group with similar acidic pH. S.S. Saline solution; i.p. intraperitoneal administration. (B) 0.5% acetic acid by i.p. administration was analyzed as positive control group producing nociceptive effect, which did not modify the licking time in the formalin test.
