# Supplementary material for: Synergistic Interaction in the Analgesic-Like Effects of Maqui Berry and Citrus Is Antagonized by Sweeteners
Source: Nutrients. 2021 Jul 19;13(7):2466. doi: 10.3390/nu13072466 (PMC8308574; doi:10.3390/nu13072466)
Supplement: Supplementary file 1 [file nutrients-13-02466-s001.zip › nutrients-1228884-supplementary.pdf]

Supplementary material

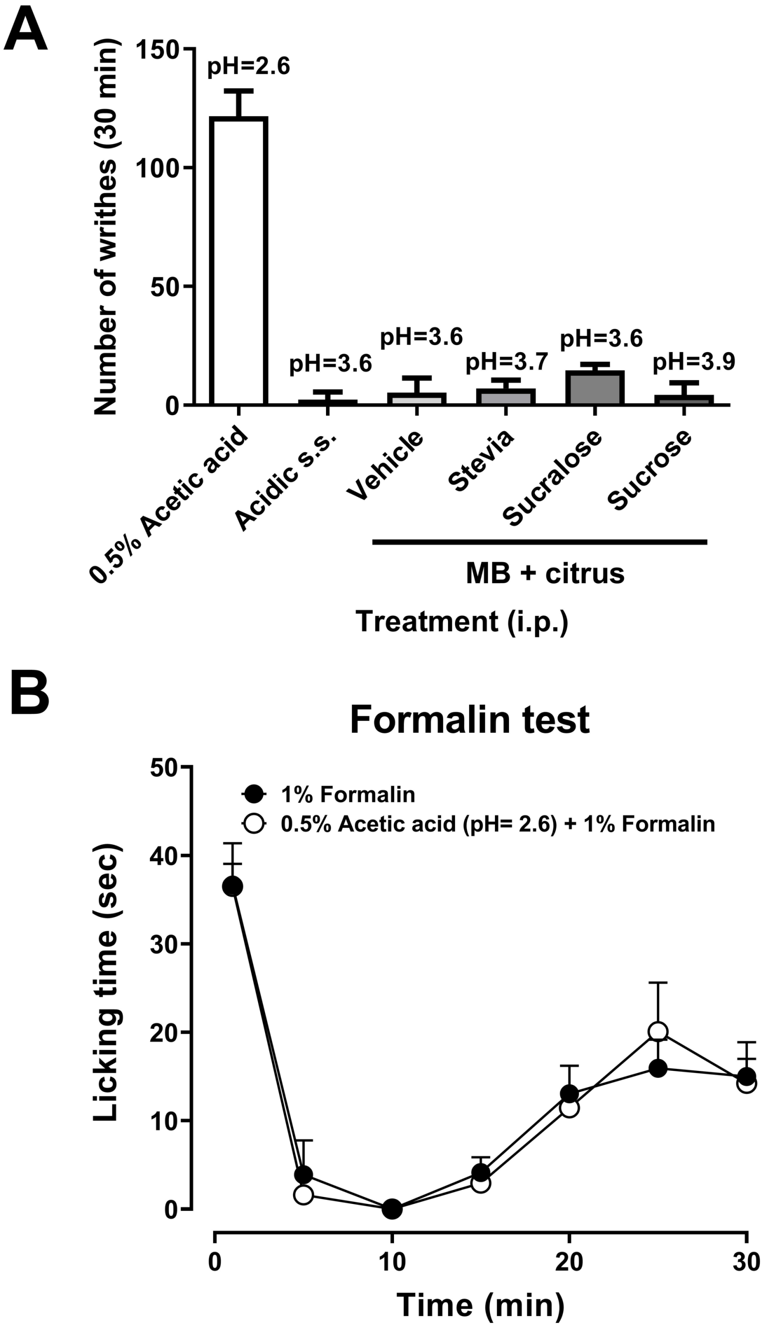

Figure S1. (A) $F_{5,12} = 194.3$, $p<0.0001$ comparison of all the treatments vs 0.5% acetic acid group in writhing behavior. Acidic s.s. as a control group with similar acidic pH. S.S. Saline solution; i.p. intraperitoneal administration. **(B)** 0.5% acetic acid by i.p. administration was analyzed as positive control group producing nociceptive effect, which did not modify the licking time in the formalin test. [35].
